# Supplementary material for: Responses of intended and unintended receivers to a novel sexual signal suggest clandestine communication
Source: Nat Commun. 2021 Feb 4;12:797. doi: 10.1038/s41467-021-20971-5 (PMC7862365; doi:10.1038/s41467-021-20971-5)
Supplement: Supplementary file 1 — Supplementary Information [file 41467_2021_20971_MOESM1_ESM.pdf]

Supplementary Information

Responses of intended and unintended receivers to a novel sexual signal suggest clandestine communication

Robin M. Tinghitella, E. Dale Broder, James H. Gallagher, Aaron W. Wikle, David M. Zonana

Supplemental Table of contents (only for items contained within this file):

Supplementary Figure 1

Supplementary Figure 2

Supplementary Table 1

Supplementary Table 2

Supplementary Table 3

Tinghitella et al. Supplementary Fig. 1 from “Responses of intended and unintended receivers to a novel sexual signal suggest clandestine communication”

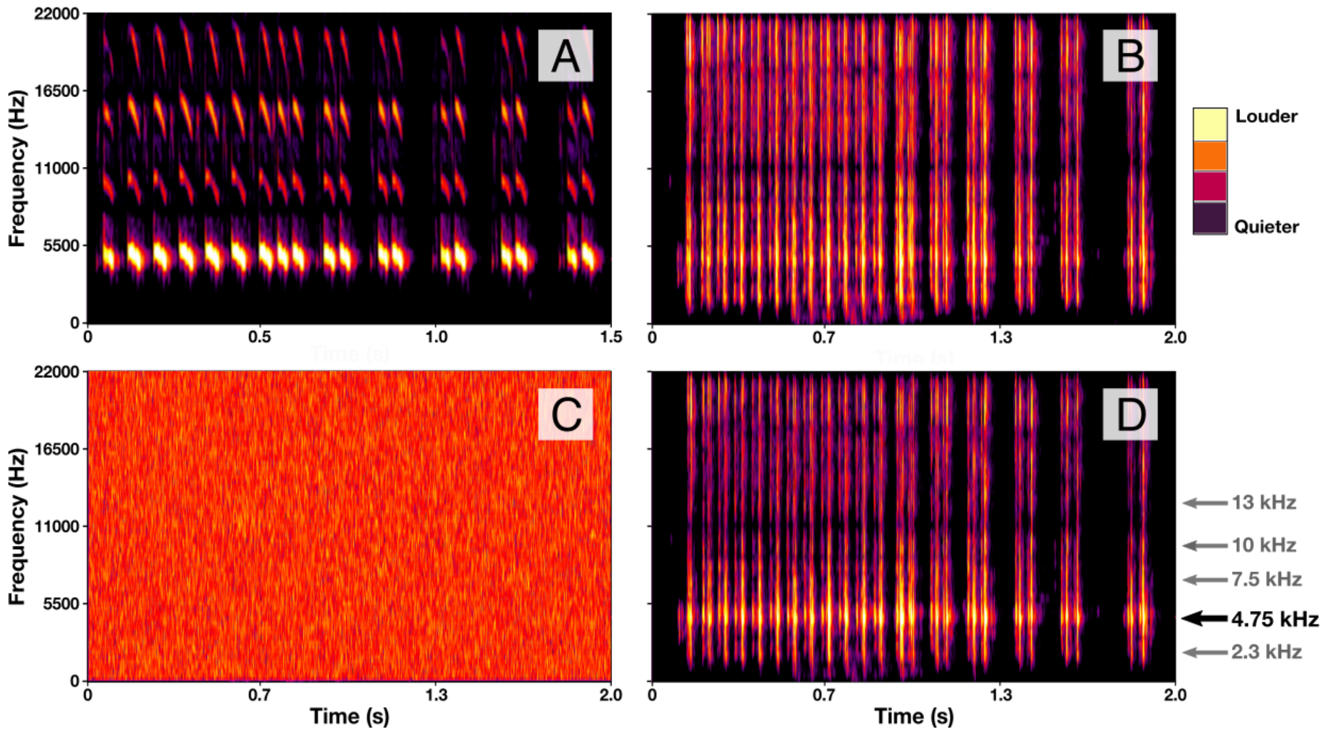

Supplementary Figure 1: Spectrograms of an ancestral song (A), an unmanipulated purring song (B), white noise (C), and a frequency manipulated song with 4.75 kHz boosted (D). The gray arrows on D also indicate the other four frequencies that were boosted in the frequency manipulation experiment.

Tinghitella et al. Supplementary Fig. 2 from "Responses of intended and unintended receivers to a novel sexual signal suggest clandestine communication"

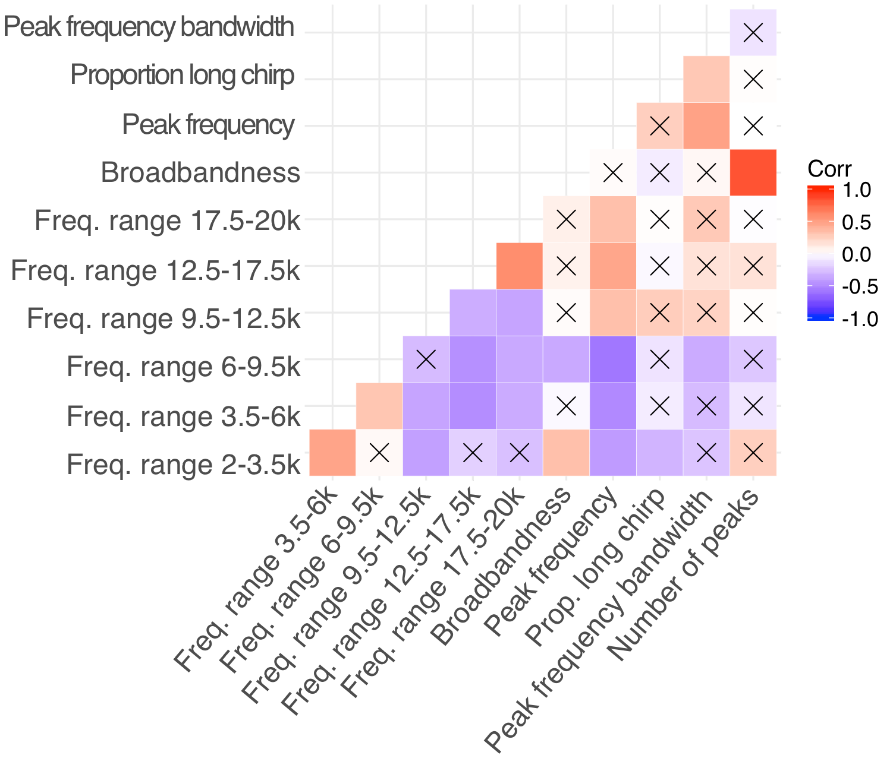

Supplementary Figure 2: Correlation matrix of purring song characteristics used in PCA. Traits with pairwise comparisons marked with an X are not significantly correlated with one another.

Tinghitella et al. Supplementary Table 1 from “Responses of intended and unintended receivers to a novel sexual signal suggest clandestine communication”

Supplementary Table 1: Frequency experiment: Outputs from linear and generalized linear mixed models assessing the influence of frequency on female cricket responses to purring songs (N = 120 female crickets). Models allowed for random intercepts for individual nested within population. Source data are provided as a Source Data file.

**Model**	**Fixed effects**			**Random effects**			
**Parameter**	**Beta**	**95% CI**	**P-value**	**N**	**SD**	**ICC**	**P-value**
Distance traveled							
(Intercept)	55.828	39.065, 72.386	**0.003**				
Frequency	-0.472	-5.345, 4.400	0.849				
Frequency^2	0.553	-5.184, 6.289	0.850				
Individual:Population				120	40.71	0.303	**<0.0001**
Population				4	11.28	0.023	0.428
Phonotaxis							
(Intercept)	-1.851	-2.467, -1.273	**<0.0001**				
Frequency	-0.165	-0.432, 0.096	0.219				
Frequency^2	-0.233	-0.540, 0.062	0.128				
Individual:Population				120	0.639	0.108	**0.030**
Population				4	0.309	0.025	0.236
Contact with speaker							
(Intercept)	-2.395	-2.989, -1.879	**<0.0001**				
Frequency	-0.186	-0.491, 0.109	0.221				
Frequency^2	-0.135	-0.486, 0.203	0.440				
Individual:Population				120	0.775	0.154	0.104
Population				4	<0.0001	NA	NA

Tinghitella et al. Supplementary Table 2 from “Responses of intended and unintended receivers to a novel sexual signal suggest clandestine communication”

Supplementary Table 2: Frequency experiment: Outputs from generalized linear mixed models assessing the influence of frequency on fly responses to purring songs (N = 37). Models allowed for random intercepts for individuals. P-values of fixed and random effects estimated with Type II Wald Chi Square tests and likelihood-ratio tests, respectively. Source data are provided as a Source Data file.

**Model**	**Fixed effects**			**Random effects**			
**Parameter**	**Beta**	**95% CI**	**P-value**	**N**	**SD**	**ICC**	**P-value**
Movement							
(Intercept)	-0.909	-2.731, 0.827	0.288				
Frequency	0.270	-0.217, 0.778	0.260				
Frequency^2	-0.021	-0.055, 0.010	0.165				
Individual				37	1.957	0.538	**<0.0001**
Contact with speaker							
(Intercept)	-7.768	-14.773, -3.346	**0.003**				
Frequency	-0.598	-1.985, 0.625	0.352				
Frequency^2	0.037	-0.042, 0.125	0.374				
Individual				37	11.57	0.976	**<0.0001**

Tinghitella et al. Supplementary Table 3 from "Responses of intended and unintended receivers to a novel sexual signal suggest clandestine communication"

Supplementary Table 3: Exemplar experiment: Effect of variation in purring song characteristics on the responses of female *T. oceanicus* (N = 271) as measured by phonotactic behavior (yes/no), contact with the speaker (yes/no) and distance traveled. Outputs from complete second-order mixed effects models (LMM: Distance; GLMM with binomial errors: Phonotaxis, Contact) using coordinates of exemplar songs along the first two PCA axes as predictor variables (PC1, PC2). Models allowed for random intercepts for individual nested within population. P-values of fixed and random effects estimated with Type III Wald Chi Square tests and likelihood-ratio tests, respectively.

**Model**	**Fixed effects**			**Random effects**			
**Parameter**	**Beta**	**95% CI**	**P-value**	**N**	**SD**	**ICC**	**P-value**
Phonotaxis							
(Intercept)	-1.915	-2.479, -1.372	**<0.0001**				
PC1	0.113	-0.005, 0.231	0.062				
PC2	-0.021	-0.147, 0.102	0.743				
PC1^2	0.081	-0.052, 0.214	0.234				
PC2^2	-0.009	-0.053, 0.035	0.690				
PC1:PC2	0.032	-0.051, 0.117	0.451				
Individual:Population				271	0.777	0.155	**<0.0001**
Population				6	0.062	0.001	0.846
Contact							
(Intercept)	-2.638	-3.388, -2.151	**<0.0001**				
PC1	0.085	-0.068, 0.240	0.278				
PC2	0.017	-0.148, 0.176	0.840				

PC1^2	0.081	-0.088, 0.253	0.350				
PC2^2	-0.032	-0.090, 0.025	0.274				
PC1:PC2	0.039	-0.069, 0.148	0.484				
Individual:Population				270	0.660	0.116	**0.0002**
Population				6	0.193	0.010	0.235
Distance							
(Intercept)	39.542	27.894, 5.230	**<0.0001**				
PC1	0.538	-1.120, 2.196	0.525				
PC2	-0.658	-2.400, 1.083	0.459				
PC1^2	1.506	-0.362, 3.373	0.115				
PC2^2	-0.317	-0.939, 0.306	0.319				
PC1:PC2	0.868	-0.317, 2.052	0.152				
Individual:Population				270	0.664	0.269	**<0.0001**
Population				6	0.190	0.051	**0.0005**
