# Supplementary material for: Responses of intended and unintended receivers to a novel sexual signal suggest clandestine communication
Source: Nat Commun. 2021 Feb 4;12:797. doi: 10.1038/s41467-021-20971-5 (PMC7862365; doi:10.1038/s41467-021-20971-5)
Supplement: Supplementary file 3 — Description of Additional Supplementary Files [file 41467_2021_20971_MOESM3_ESM.pdf]

Description of Additional Supplementary Files

File Name: Supplementary Data 1
Description: Data and data README accompanying "Responses of intended and unintended receivers to a novel sexual signal suggest clandestine communication"
