# Supplementary material for: Responses of intended and unintended receivers to a novel sexual signal suggest clandestine communication
Source: Nat Commun. 2021 Feb 4;12:797. doi: 10.1038/s41467-021-20971-5 (PMC7862365; doi:10.1038/s41467-021-20971-5)
Supplement: Supplementary file 4 — Supplementary Data 1 [file 41467_2021_20971_MOESM4_ESM.zip › Supplementary_Data1/Tinghitella_etal_SuppData_README.pdf]

README for data accompanying “Responses of intended and unintended receivers to a novel sexual signal suggest clandestine communication”

David Zonana

12/18/2020

“T.oceanicus_frequency_manipulation_experiment.csv”

This file contains data on responses of female *T. oceanicus* from the frequency manipulation experiment. The data contains 6 columns:

- $uniqueID: a unique identifier for individual females. For each female, the ID is composed of the population name, numeric ID, and sampling trip, separated by underscores (population.name_female.numeric.ID_sampling.trip).
- $Date: date of phonotaxis trial.
- $Frequency: the song played in each observation. Songs with numeric values (e.g. 2.3) represent manipulated purring songs with amplitudes boosted in the stated frequency range.
- $Phonotaxis: binary response of whether or not a female exhibited phonotactic behavior, as described in the manuscript.
- $Distance: the maximimum distance (cm) traveled by a female from the starting point.
- $Contact: binary response of whether or not a female contacted the speaker.

“O.ochracea_frequency_manipulation_experiment.csv”

Responses of female *Ormia ochracea* from frequency manipulation experiment. The data contains 5 columns:

- $uniqueID: a unique identifier for individual females. For each female, the ID is composed of the population name, numeric ID, and sampling trip, separated by underscores (population.name_female.numeric.ID_sampling.trip).
- $Date.tested: date of phonotaxis trial.
- $Frequency: the song played in each observation. Songs with numeric values (e.g. 2.3) represent manipulated purring songs with amplitudes boosted in the stated numeric range.
- $Movement: binary response of whether or not the fly made any movement towards the speaker.
- $Contact: binary response of whether or not a female contacted the speaker.

"T.oceanicus_exemplar_experiment.csv"

This file contains data on responses of female *T. oceanicus* from the exemplar experiment. The file contains 10 columns:

- $uniqueID: a unique identifier for individual females. For each female, the ID is composed of the population name, numeric ID, and sampling trip, separated by underscores (population.name_female.numeric.ID_sampling.trip).
- $Population: the female's population of origin.
- $Year: the sampling trip on which data was collected (S19 refers to 'summer of 2019' and W19.20 refers to the 'winter of 2019/2020')
- $Date: date of phonotaxis trial.
- $song: the song played in each observation. Songs E1-8 represent the eight exemplars of purring songs used in the experiment. Trials coded as LOUD are a purring exemplar played at equivalent amplitude as the typical *T.oceanicus* calling song.
- $song_simple: same as song, but all exemplars simply coded as "purrs".
- $Phonotaxis: binary response of whether or not a female exhibited phonotactic behavior, as described in the manuscript.
- $Distance: the maximimum distance (cm) traveled by a female from the starting point.
- $Time: for females that did contact the speaker, the number of seconds needed to make contact.
- $Contact: binary response of whether or not a female contacted the speaker.

"O.ochracea_trapping_data.csv"

Field data of *Ormia ochracea* trapping success from triangular arrays of sound-funnel traps. In each triangle, one speaker broadcast typical *T. oceanicus* calling song, one a purring *T. oceanicus* song, and the third a looped track of white noise. The file contains 5 columns:

- $date: date of trapping attempt.
- $population: population where trap was set.
- $triangle: a number representing which triangle array replicate a trap was part of.
- $song: the song that the funnel trap was broadcasting.
- $flies: the number of flies present in the traps after the entirety of a trapping session.

"purring_song_characteristics.csv"

A data set of acoustic song characteristics from 46 recorded, purring *T. oceanicus* males. The file contains 14 columns:

- $Population: the male's population of origin.
- $Male.ID: a male's numeric ID.
- $exemplar: recordings that were used as exemplars are noted (e.g. E1, E2, E3…). Recordings that were used in the PCA, but were not chosen as exemplars contain NA values.

- $relAmp… (6 of these columns): dB difference between each defined frequency range and the song's total amplitude. Frequency ranges include: 2-3.5, 3.5-6, 6-9.5, 9.5-12.5, 12.5-17.5, 17.5-20 kHz. Ranges determined based on cricket hearing ability (Imaizumi et al. 1999).
- $Broadbandness: Range (in Hz) of frequency bands 10 dB below the peak frequency band's apex.
- $Peak.Frequency: Frequency with the greatest acoustic power.
- $Prop.Long.Chirp: Length of the long chirp (in ms) divided by the sum of the long and short chirp lengths.
- $Peak.Frequency.Bandwidth: Difference between upper and lower frequencies 10 dB below the peak frequency band's apex.
- $Number.of.Peaks: Number of frequency bands between 1.5 and 20 kHz that fall within 10 dB of the peak frequency band's apex.
