# Supplementary material for: Responses of intended and unintended receivers to a novel sexual signal suggest clandestine communication
Source: Nat Commun. 2021 Feb 4;12:797. doi: 10.1038/s41467-021-20971-5 (PMC7862365; doi:10.1038/s41467-021-20971-5)
Supplement: Supplementary file 5 — Reporting Summary [file 41467_2021_20971_MOESM5_ESM.pdf]

Corresponding author(s): Tinghitella, Robin

Last updated by author(s): Dec 17, 2020

Reporting Summary

Nature Research wishes to improve the reproducibility of the work that we publish. This form provides structure for consistency and transparency in reporting. For further information on Nature Research policies, see our Editorial Policies and the Editorial Policy Checklist.

Statistics

For all statistical analyses, confirm that the following items are present in the figure legend, table legend, main text, or Methods section.

n/a	Confirmed	
☐	☒	The exact sample size (*n*) for each experimental group/condition, given as a discrete number and unit of measurement
☐	☒	A statement on whether measurements were taken from distinct samples or whether the same sample was measured repeatedly
☐	☒	The statistical test(s) used AND whether they are one- or two-sided<br>*Only common tests should be described solely by name; describe more complex techniques in the Methods section.*
☐	☒	A description of all covariates tested
☒	☐	A description of any assumptions or corrections, such as tests of normality and adjustment for multiple comparisons
☐	☒	A full description of the statistical parameters including central tendency (e.g. means) or other basic estimates (e.g. regression coefficient) AND variation (e.g. standard deviation) or associated estimates of uncertainty (e.g. confidence intervals)
☐	☒	For null hypothesis testing, the test statistic (e.g. *F*, *t*, *r*) with confidence intervals, effect sizes, degrees of freedom and *P* value noted<br>*Give P values as exact values whenever suitable.*
☒	☐	For Bayesian analysis, information on the choice of priors and Markov chain Monte Carlo settings
☐	☒	For hierarchical and complex designs, identification of the appropriate level for tests and full reporting of outcomes
☒	☐	Estimates of effect sizes (e.g. Cohen's *d*, Pearson's *r*), indicating how they were calculated

Our web collection on statistics for biologists contains articles on many of the points above.

Software and code

Policy information about availability of computer code

Data collection	No software was used during data collection.
Data analysis	Cricket songs were analyzed and manipulated in Audacity (version 2.3.1) and Logic Pro X (version 10.4.8). Data analysis was completed in R (version 3.6.0) using the following packages: lme4 (Bates et al. 2015), PFunc (Kilmer et al. 2017), performance (Ludecke et al. 2020), lmerTest (Kuznetsova et al. 2017), caret (Kuhn 2008), and mgcv (Wood 2003).

For manuscripts utilizing custom algorithms or software that are central to the research but not yet described in published literature, software must be made available to editors and reviewers. We strongly encourage code deposition in a community repository (e.g. GitHub). See the Nature Research guidelines for submitting code & software for further information.

Data

Policy information about availability of data

All manuscripts must include a data availability statement. This statement should provide the following information, where applicable:

- Accession codes, unique identifiers, or web links for publicly available datasets
- A list of figures that have associated raw data
- A description of any restrictions on data availability

The authors declare that the data supporting the findings in this study are available within this paper and the as Supplementary Data 1 file. Source data for Figures 2-5 and all Tables are also provided with the paper.

Field-specific reporting

Please select the one below that is the best fit for your research. If you are not sure, read the appropriate sections before making your selection.

☐ Life sciences ☐ Behavioural & social sciences ☒ Ecological, evolutionary & environmental sciences

For a reference copy of the document with all sections, see nature.com/documents/nr-reporting-summary-flat.pdf

Ecological, evolutionary & environmental sciences study design

All studies must disclose on these points even when the disclosure is negative.

Study description	In this study we test responses of conspecific crickets (intended receivers) and parasitoid flies (unintended receivers) to a novel sexual signal (purring) in six replicate cricket populations across the Hawaiian archipelago. We conducted three complementary studies: 1) testing responses of individuals to categorical song differences (ancestral, purring, and a white noise negative control), 2) characterizing selection acting on purring song by measuring responses of individuals to purring songs manipulated to differ in frequency (pitch) only, and 3) characterizing selection on the novel purring song by measuring responses of individuals to natural purring songs that differ in multiple song characteristics.
Research sample	Adult female Teleogryllus oceanicus and Ormia ochracea from six Hawaiian populations: Laie, Oahu; Manoa, Oahu; Hilo, Hawaii; Kalaupapa, Molokai; Wailua, Kauai; Kapaa, Kauai. Sites were chosen because the two species co-occur in these locations and different morphs of T. oceanicus (e.g., ancestral, purring, silent) occur in different combinations across the locations.
Sampling strategy	We search for T. oceanicus exhaustively at well-established and long-studied field sites, although, as with all field studies, sample sizes are limited by animal abundance. We aimed to test a minimum of 30 individuals per population in each experiment, as is common in behavioral preference studies (range = 22-44 females per population in the Frequency Manipulation experiment and 18 - 60 females per population in the Exemplar Experiment).
Data collection	All co-authors collected and recorded data during field-based fly phonotaxis tests and no-choice cricket phonotaxis tests by direct observation. Timing was done using stopwatches. We recorded data live and directly into web-based google spreadsheets. Two to three trained observers came to agreement on all dependent behavioral variables before recording the data in the spreadsheet (distance traveled, phonotaxis, and contact) in phonotaxis tests, while an additional researcher randomized the order of song playbacks, timed trials using the stopwatch, and recorded data reported by the behavioral observers.
Timing and spatial scale	Neither study species is seasonal in the Hawaiian portion of their range. We sampled at approximately six month intervals to avoid pseudoreplication, with all experiments conducted between June 2018 and January 2020. The parasitoid fly field experiment was conducted in June 2019 and Dec 2019 - Jan 2020, the Frequency Manipulation experiment was conducted in June 2018 and Dec 2018-Jan 2019, and the Exemplar experiment was conducted in June 2019 and Dec-Jan 2020. All sites within a given experiment were sampled on each sampling trip.
Data exclusions	The only animals eliminated from analyses were female crickets that did not respond positively to ANY song playbacks (including positive controls). Females that eclosed to adulthood recently (<72 hours) do not exhibit phonotactic behaviors. This is stated explicitly in the manuscript.
Reproducibility	These experiments were conducted across four trips to the field sites in Hawaii between June 2018 - January 2020. Patterns were qualitatively similar across trips, across two large-scale phonotaxis experiments (the Frequency Manipulation Experiment and Exemplar Experiment), and robust to different statistical approaches.
Randomization	In parasitoid fly field studies, we randomized the location of playbacks within each field site and also the location of songs within replicates. All crickets tested in phonotaxis tests were tested with each song model and the order of presentation of song stimuli was random, with the exception of the ancestral song positive control, which was played last because it tends to be the most stimulating song and could alter responses to other songs.
Blinding	The data recorder randomized which song was played back during phonotaxis trials independent of the behavioral observers. While behavioral observers could hear the songs played, variation in the ranges of song characteristics tested are not easily discernible by human ears. Additionally, two to three trained observers came to consensus on all behavioral variables recorded.

Did the study involve field work? ☒ Yes ☐ No

Field work, collection and transport

Field conditions	Fortunately our field sites are located in a region with little temperature variation and no choice phonotaxis tests were conducted indoors at field stations with moderated temperatures (~26 C). When extreme weather events like strong winds and rainfall occurred, we did not conduct field-based parasitoid fly experiments (as these conditions impede fly activity).
Location	Hilo: 19.701211209874117, -155.08143357152687, 59’<br>Kalaupapa: 221.189754808759396, -156.9817700133905, 66’<br>La'ie: 21.643523369214055, -157.92452111967387, 9’<br>Wailua: 22.067246714503696, -159.39605354575824, 532’<br>Kapa’a: 22.073373744153866, -159.318867445758, 20’

	Manoa: 21.31235536376053, -157.81120501145728, 95'
Access & import/export	While we do hold active USDA import/export permits for Hawaiian T. oceanicus (P526P-19-04265), all of the experiments reported here were conducted on site at the field locations listed above. We work with land owners for permission to collect at several sites and with station directors at the Kauai Agricultural Station. Permits from the National Park Service (KALA-2017-SCI-0002, KALA-2018-SCI-0005) supported the work at Kalaupapa National Historical Site.
Disturbance	All tested animals were returned directly to the fields from which they were collected immediately after the experiments, minimizing disturbance to natural populations.

Reporting for specific materials, systems and methods

We require information from authors about some types of materials, experimental systems and methods used in many studies. Here, indicate whether each material, system or method listed is relevant to your study. If you are not sure if a list item applies to your research, read the appropriate section before selecting a response.

Materials & experimental systems

n/a	Involved in the study
☒	☐ Antibodies
☒	☐ Eukaryotic cell lines
☒	☐ Palaeontology and archaeology
☐	☒ Animals and other organisms
☒	☐ Human research participants
☒	☐ Clinical data
☒	☐ Dual use research of concern

Methods

n/a	Involved in the study
☒	☐ ChIP-seq
☒	☐ Flow cytometry
☒	☐ MRI-based neuroimaging

Animals and other organisms

Policy information about studies involving animals; ARRIVE guidelines recommended for reporting animal research

Laboratory animals	The study did not involve laboratory animals.
Wild animals	We hand captured adult female Teleogryllus oceanicus and trapped Ormia ochracea (using sound funnel traps) in six Hawaiian populations: Laie, Oahu; Manoa, Oahu; Hilo, Hawaii; Kalaupapa, Molokai; Wailua, Kauai; Kapaa, Kauai. All animals were released immediately after behavioral studies into the same field from which they were collected.
Field-collected samples	Collected crickets were maintained in standard 15L totes with cover, moist cotton, and ad lib food on a natural light:dark cycle for a maximum of four days. Collected flies were held in butterfly flight cages with nectar and structure under a natural light:dark cycle for a maximum of two days. At the end of the experiments all animals were returned directly to the fields from which they were collected originally.
Ethics oversight	No ethical approval was required as the animals are not covered by IACUC.

Note that full information on the approval of the study protocol must also be provided in the manuscript.
